# Can Neurotypical Individuals Read Autistic Facial Expressions? Atypical Production of Emotional Facial Expressions in Autism Spectrum Disorders

**DOI:** 10.1002/aur.1508

**Published:** 2015-06-06

**Authors:** Rebecca Brewer, Federica Biotti, Caroline Catmur, Clare Press, Francesca Happé, Richard Cook, Geoffrey Bird

**Affiliations:** ^1^ MRC Social, Genetic and Developmental Psychiatry CentreInstitute of Psychiatry, Psychology and Neuroscience, King's College London; ^2^ Department of PsychologyCity University, London; ^3^ School of Psychology, University of Surrey; ^4^ Department of Psychological SciencesBirkbeck College London; ^5^ Institute of Cognitive Neuroscience, University College London (G.B.)

**Keywords:** social cognition, face perception, cognitive neuroscience, expression production

## Abstract

The difficulties encountered by individuals with autism spectrum disorder (ASD) when interacting with neurotypical (NT, i.e. nonautistic) individuals are usually attributed to failure to recognize the emotions and mental states of their NT interaction partner. It is also possible, however, that at least some of the difficulty is due to a failure of NT individuals to read the mental and emotional states of ASD interaction partners. Previous research has frequently observed deficits of typical facial emotion recognition in individuals with ASD, suggesting atypical representations of emotional expressions. Relatively little research, however, has investigated the ability of individuals with ASD to produce recognizable emotional expressions, and thus, whether NT individuals can recognize autistic emotional expressions. The few studies which have investigated this have used only NT observers, making it impossible to determine whether atypical representations are shared among individuals with ASD, or idiosyncratic. This study investigated NT and ASD participants’ ability to recognize emotional expressions produced by NT and ASD posers. Three posing conditions were included, to determine whether potential group differences are due to atypical cognitive representations of emotion, impaired understanding of the communicative value of expressions, or poor proprioceptive feedback. Results indicated that ASD expressions were recognized less well than NT expressions, and that this is likely due to a genuine deficit in the representation of typical emotional expressions in this population. Further, ASD expressions were equally poorly recognized by NT individuals and those with ASD, implicating idiosyncratic, rather than common, atypical representations of emotional expressions in ASD. ***Autism Res*** *2016, 9: 262–271*. © 2015 International Society for Autism Research, Wiley Periodicals, Inc.

## Introduction

Autism spectrum disorder (ASD) is characterized by impaired communication and social interaction, and restricted and repetitive interests [American Psychiatric Association, 2013]. Although not required for diagnosis, it is often assumed that social interaction atypicalities stem, in part, from deficits of emotion recognition [Harms, Martin, & Wallace, 2010]. Emotion processing atypicalities would be detrimental to social interactions, where correct recognition of the emotional state of one's interaction partner allows for an appropriate response. While evidence for emotion recognition deficits in ASD has been equivocal [Harms et al., 2010], it is still the case that numerous studies have observed atypical recognition of others’ emotions in this population [Ashwin, Chapman, Colle, & Baron‐Cohen, 2006; Dziobek, Bahnemann, Convit, & Heekeren, 2010; Greimel et al., 2010; Lindner & Rosén, 2006]. Recent evidence suggests that co‐occurring alexithymia, associated with atypical recognition of others’ facial emotion [Grynberg et al., 2012; Jessimer & Markham, 1997; Lane et al., 1996; McDonald & Prkachin, 1990; Swart, Kortekaas, & Aleman, 2009] is, in fact, responsible for emotion recognition deficits in these individuals [Cook, Brewer, Shah, & Bird, 2013]. However, the fact that alexithymia is highly prevalent in ASD, with approximately 50% of individuals meeting criteria for severe levels of alexithymia [Berthoz & Hill, 2005; Hill, Berthoz, & Frith, 2004], makes emotion recognition problematic for many individuals with ASD, despite not being a necessary feature of the condition. Many individuals with ASD are, therefore, likely to suffer deficits of facial emotion recognition, which, in turn, impair social interaction.

It is clear that difficulties recognizing the emotions of an interaction partner may reduce the quality of social interactions, but little research has acknowledged the fact that this is necessarily a bi‐ (or multi‐) directional process [Halberstadt, Denham, & Dunsmore, 2001]; interactions involve more than one individual, and social cognition may vary depending on our engagement in that interaction [Schilbach et al., 2013]. Individuals with ASD may be poor at reading neurotypical (NT) facial expressions, but NT individuals may also be impaired at reading ASD expressions. Both recognition and production of emotional facial expressions rely on internal representations of the underlying physical components of each expression. During interactions, successful conveyance of emotion relies on the individuals involved sharing common representations of emotions; when trying to interpret emotional signals, one must compare the physical features of the observed expression to those of one's own internal representations of expressions. Similarly, when attempting to convey a particular emotion, one must produce an expression which matches the representation of that emotion held by one's interaction partner. A mismatch between “sender” and “receiver” in underlying representations for emotional expression would, therefore, lead to a failure to communicate emotion [see also Cook, Blakemore, & Press, 2013]. It is possible that past testing of ASD emotion recognition, using solely NT expressions, has resulted in an underestimation of ASD capabilities; ASD individuals may be better able to read the emotional expressions of ASD “senders”.

Given documented (although not universal) difficulties in ASD in recognizing (NT) emotional expression, it is important to know whether ASD individuals can send clear emotional signals. There has been surprisingly little experimental research on emotional expression differences in ASD [Begeer, Koot, Rieffe, Meerum Terwogt, & Stegge, 2008]. Previous evidence suggests atypical use of nonverbal expressive behaviors in children with ASD in naturalistic settings [Bieberich & Morgan, 2004; Capps, Kasari, Yirmiya, & Sigman, 1993; Dawson, Hill, Spencer, Galpert, & Watson, 1990; Kasari, Sigman, Mundy, & Yirmiya, 1990; Snow, Hertzig, & Shapiro, 1887; Stagg, Slavny, Hand, Cardoso, & Smith, 2014], reduced facial muscle movements during play situations [Czapinski & Bryson, 2003], and “awkward” facial expressions of emotion during emotional story‐telling [Grossman, Edelson, & Tager‐Flusberg, 2013]. In more explicit tasks of expression posing, observational evidence suggests that children with ASD struggle to communicate happiness using facial expressions [Langdell, 1981].

While evidence is mounting that suggests individuals with ASD may express affect atypically, few studies have specifically investigated the ability of these individuals to produce emotional facial expressions that are recognizable by others. Macdonald et al. [1989] previously observed, in a sample of 10 high‐functioning adults with ASD, impaired ability to express facial emotion in a way that could be correctly interpreted by NT judges. More recently, Volker, Lopata, Smith, and Thomeer [2009] found in a larger sample of children that NT raters were less able to recognize expressions of sadness when produced by children with ASD than by typically developing children. ASD expressions were also judged to be more “odd” than NT expressions. Faso et al. [2014] similarly found that NT judges perceived the facial expressions of adults with ASD to be less natural, and more intense, than of those without ASD. Surprisingly, however, ASD emotion expressions of anger were better recognized, and expressions of happiness worse recognized, than NT expressions.

While these findings highlight atypical expression production, which could be highly detrimental to the social functioning of individuals with ASD, previous studies are limited by small numbers of participants expressing emotions, or raters attempting to recognize them (typically 5 or 6 raters). Crucially, all studies have included only NT individuals as raters. Just as employing NT facial expressions in recognition tasks may be problematic, employing NT raters in expression tasks may disadvantage ASD participants; individuals with ASD may struggle to interact with NT individuals partially due to problems interpreting typical expressions of facial emotion, but also due to NT individuals finding it difficult to interpret autistic expressions of emotion. If ASD is associated with atypical emotion representations, two possibilities exist; individuals with ASD may have idiosyncratic representations (varying between ASD individuals), or they may share common, but atypical (varying from NT individuals), representations. If the latter is true, it is possible that those with ASD are able to recognize the emotional facial expressions of others with ASD, but not of NT individuals; emotional expressions of ASD participants in previous studies may have been correctly interpreted by autistic raters. The consistent use of NT individuals on one side of the artificial “interaction” in previous tasks may, therefore, have placed individuals with ASD at a disadvantage.

Whether atypicalities, should they exist, are systematic or idiosyncratic in ASD samples is an important question, as it has strong implications for ASD sociality and the way in which interventions are delivered. If ASD expressions vary systematically from typical expressions, individuals with ASD may be able to interact with other individuals with ASD without emotion recognition difficulties, while interactions between ASD and typical individuals may be characterized by expression recognition difficulties on both sides. If ASD expressions are idiosyncratic, however, individuals with ASD may struggle to interpret the expressions of all other individuals, as well as express emotions in a way others can understand, regardless of whether the observer has ASD or not. Similarly, the existence of systematic variations would support the use of standard interventions for the production of typical (and therefore, more recognizable) expressions, as the necessary adjustments would be consistent across individuals. Interventions for idiosyncratic expression production, conversely, would require each individual's variation from typical expressions to be quantified and incorporated into the intervention.

Previous studies of emotional expression in ASD were unable to determine the extent to which expression atypicalities were due to atypical representation of emotional information, or other confounded factors. While unusual expressions may reflect atypical cognitive representations of facial emotion (one's internal depiction of the physical attributes of each expression), consistent with impaired emotion recognition in some individuals with ASD, alternative explanations remain. Decreased awareness about the communicative value of facial expressions, or reduced motivation to transfer this information, for example, could also cause atypical expressions. Similarly, individuals with ASD may have reduced proprioceptive awareness of facial muscle movements [Weimer, Schatz, Lincoln, Ballantyne, & Trauner, 2001]. Impaired feedback information concerning one's facial movements, or reduced ability to process this information, may lead to atypical production of expressions simply due to motor output being poorly matched to internal representations of emotion, rather than due to atypical representations themselves.

This study tested the hypothesis that individuals with ASD produce atypical emotional expressions, due to atypical representations of facial emotion. In order to determine whether these representations differ systematically or idiosyncratically from NT expressions, the ability of participants with and without ASD to recognize these expressions was investigated. Finally, three posing conditions allowed for distinction between atypical representations, communication awareness or motivation, and proprioceptive awareness. These conditions involved participants posing facial emotions naturally, posing expressions with the aim of enabling the experimenter to correctly guess the emotion, and posing expressions with visual feedback available. Use of these conditions allowed the nature of any expression production deficit to be investigated; if global representational problems exist in ASD, poorly recognizable expressions would be produced in all conditions, while communication awareness or proprioception problems should result in an interaction between poser group and posing condition (ASD expressions being poorly recognized when produced in the standard posing condition, but not in the communicative and visual feedback conditions, respectively). As previous findings suggest that alexithymia can account for atypicalities in emotion recognition, alexithymia was measured and taken into account both in participants posing and recognizing emotional expressions.

## Method

### Stimulus Development

#### Participants

Sixteen adults with ASD (3 females) and 17 NT individuals, with no clinical diagnosis (2 females) participated in stimulus development for this study. ASD and control groups were matched according to age [*t*(31) = 1.911, *P* = 0.065], gender [*X* ^2^ = 0.480, *P* = 0.489] and IQ [*t*(31) = 0.837, *P* = 0.409], measured by the Wechsler Abbreviated Scale of Intelligence [WASI; Wechsler, 1999] and Wechsler Adult Intelligence Scale [WAIS; Wechsler, 1997]. Autism symptom severity was measured in all participants using the Autism‐Spectrum Quotient [AQ; Baron‐Cohen, Wheelwright, Skinner, Martin, & Clubley, 2001]. Alexithymia was measured using the Toronto Alexithymia Scale [TAS‐20; Bagby, Parker, & Taylor, 1994]. Current functioning of all individuals in the ASD group was assessed with the Autism Diagnostic Observation Schedule [ADOS; Lord et al., 2000]. ADOS scores meeting criteria for ASD may be categorized as indicative of either “autism” or “autism spectrum.” Of the 16 participants with a clinical diagnosis of ASD, 14 also met the ADOS criteria for ASD (10 for autism, 4 for autism spectrum). Although two of the individuals in the ASD group did not meet criteria for ASD, according to the ADOS, they received diagnoses from independent clinicians and one scored above cutoff for autism on the AQ.

#### Procedure

Poser participants were asked to pose the six basic emotions (happiness, sadness, fear, surprise, anger, disgust) while being recorded with a video camera. Three posing conditions were used, and were completed in a standardized order. The first condition—the standard condition—involved participants being instructed to pose each emotion to the best of their ability using facial expressions, and to express the emotions for approximately 3 sec. Participants were asked to precede and follow each emotional expression with a neutral expression. Order of emotion type was randomized across participants. This posing condition was used to determine individuals’ ability to pose expressions without the use of visual feedback, and without the communicative value of expressions being emphasized. It was intended to be the most similar to the conditions in which individuals may deliberately pose facial expressions in everyday life. The second, “communicate,” condition was included to emphasize the communicative value of emotional expressions, in order that individuals with ASD should not be disadvantaged by potentially reduced comprehension of the emotional information that can be conveyed to others using facial expressions. This condition was used to ensure that any differences between the ASD and control expressions were not simply due to individuals with ASD failing to comprehend the informative nature of facial expressions. The communicate condition required participants to randomly select each of the six emotions (written on cards) and pose it, in a way that would allow the experimenter to guess which emotion was being expressed. Following each expression, the experimenter attempted to guess which emotion was being expressed, and participants were instructed not to give any feedback until the end of the task. The order of the expressed emotions was tracked by participants placing emotion cards face down in a pile once the relevant expression had been posed, and recorded by the experimenter. The third condition—the mirror condition—was included to investigate the impact of visual feedback on participants’ expressive ability. This condition was included to ensure that any group differences were not due to individuals with ASD having reduced awareness of their facial movements. The mirror condition allowed individuals to produce the expression that best matched their internal representation of the visual features of each facial emotion, as the visual aspects of their own expression could be monitored during production. It involved participants posing the emotions while watching their expressions in a camera, which acted as a mirror. Again, order of emotion was randomized across participants.

#### Creation of static emotional facial expression stimuli

**Figure 1:**
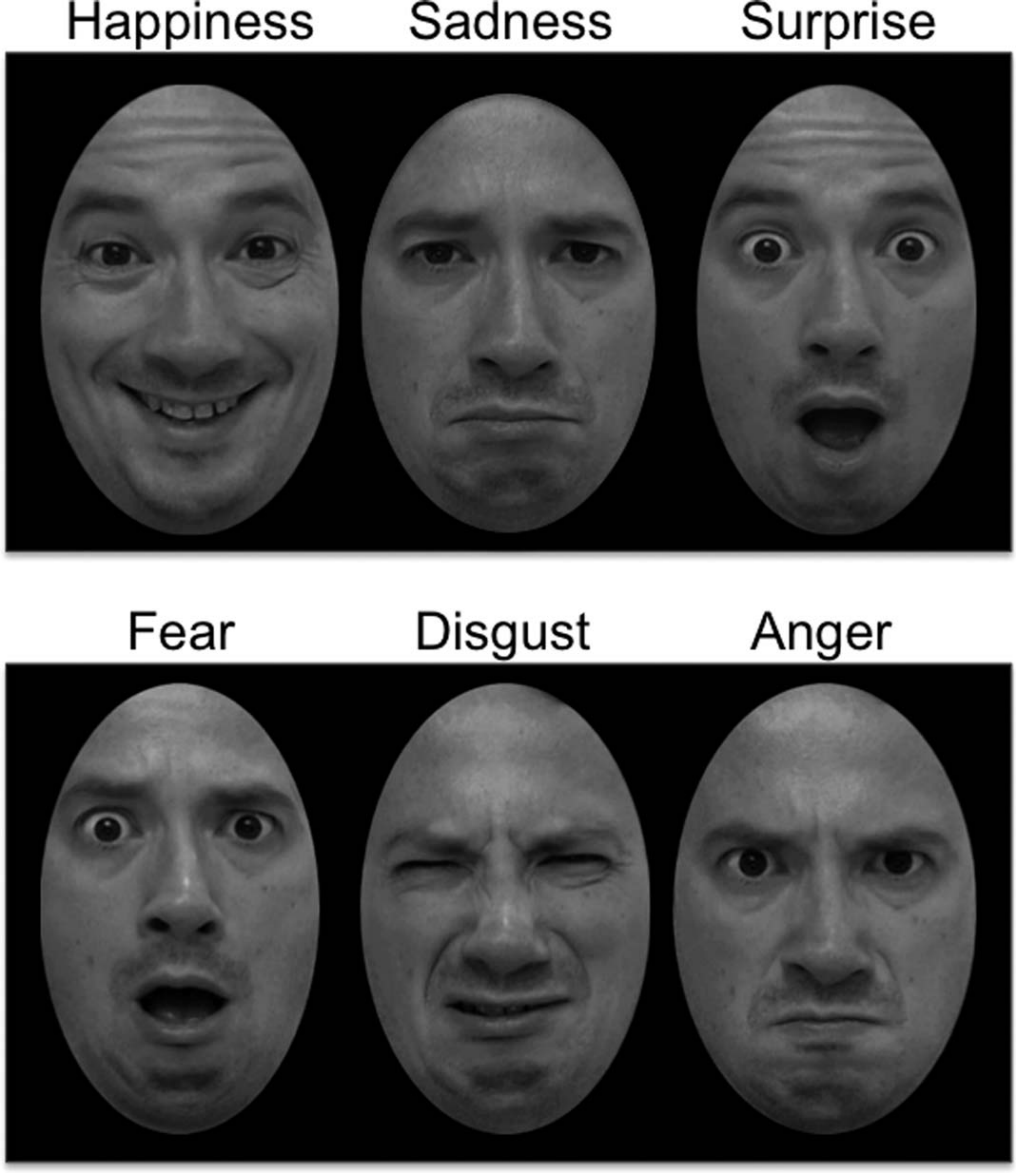
Examples of stimuli, showing all six emotions posed by one participant in the standard posing condition.

Fifteen ASD individuals (3 females) and 12 NT participants (0 female) gave consent for stimuli to be created from their video data and shown to other participants in a behavioral study. From each of these individuals, static stimuli were created depicting each emotion, expressed in each of the three posing conditions. This yielded a total of 486 stimuli. Each stimulus was a single frame selected from each expression video. Frames were selected such that they depicted the final state of the expression, before the participant relaxed into a neutral expression. Frames were independently selected by two trained raters, and where disagreements occurred, raters discussed frame selection and reached a consensus. Static expression images were then converted to grayscale, and cropped to exclude hair and external features (Fig. 1). The 486 resulting images were used in the following behavioral paradigm.

### Emotion Recognition Phase

#### Participants

Fourteen adults with ASD (1 female) and 13 control individuals (0 female) participated in this study. Of these participants, 10 ASD participants and 4 NT participants had taken part in the stimulus production stage. Participants were matched according to gender (*X* ^2^ = 0.96, *P* = 0.326) and IQ [*t*(25) = 1.68, *P* = 0.105], measured by the WAIS or WASI. The Control (*M* = 31.62, SD = 9.66) and ASD (*M* = 44.86, SD = 13.06) groups differed in age [*t*(25) = 2.98, *P* = 0.006], so age was included as a covariate in all analyses. ASD symptom severity was measured in all participants using the Autism‐Spectrum Quotient [AQ; Baron‐Cohen et al., 2001], and alexithymia was measured using the TAS‐20 [Bagby et al., 1994]. Informed consent was obtained from all participants. Again, the ADOS [Lord et al., 2000] was used to assess current functioning of all individuals in the ASD group. Of the 14 participants with a clinical diagnosis of ASD, 13 also met the ADOS criteria for ASD (10 for autism, 3 for autism spectrum). Although one of the individuals in the ASD group did not meet criteria for ASD according to the ADOS, this individual received a diagnosis from an independent clinician and scored above cutoff for autism on the AQ.

#### Stimuli and procedure

Stimuli were the 486 emotional facial images described above. These comprised 270 ASD expressions (54 females) and 216 control expressions (0 female). In order to match the number of ASD and control expressions viewed by each participant, but maximize the number of poser participants viewed, each individual taking part in the behavioral study viewed 216 ASD and 216 control expressions. Expressions posed by three ASD individuals were, therefore, removed from the total stimulus set at random for each behavioral study participant. Equal numbers of expression stimuli depicting each emotion and produced in each posing condition were viewed by all participants. Where participants had taken part in expression production, responses to their own expressions were removed from analyses.

Each trial consisted of a single expression stimulus, presented on a black background for 800 ms, followed by a six‐alternative forced choice prompt to attribute emotion to the stimulus. Participants were instructed to select the emotion that best described the expression. This was replaced by a prompt to express the degree of confidence in their choice on a scale from 1 to 9. Stimulus order was fully randomized, and participants were blind to both ASD diagnosis of posers, and the posing condition in which each stimulus was produced. The task lasted approximately 40 min and was written in Matlab (The MathWorks, Natick, MA) using the Psychophysics Toolbox [Brainard, 1997; Pelli, 1997].

## Results

Recognition accuracy was measured using percentage of correct trials, due to the inappropriateness of methods based on signal detection theory, such as *d*′, with more than two response options. Analysis of covariance, controlling for age, was performed on accuracy data (proportion of correct responses) with Poser Group (ASD, NT), Posing Condition (Standard, Communicate, Mirror) and Emotion (Happiness, Sadness, Surprise, Fear, Disgust, Anger) entered as within‐participants variables, and Recognizer Group (ASD, NT) entered as a between‐participants variable. For all post hoc comparisons, Bonferroni‐corrected statistics are reported. See Table 1 for all means and standard deviations.

**Table 1:** Means and standard deviations of accuracy (percentage correct) and confidence ratings in all conditions

Posing condition	Emotion	NT recognizer		ASD recognizer	
		NT poser Mean (SD)	ASD poser Mean (SD)	NT poser Mean (SD)	ASD poser Mean (SD)
*Accuracy*					
Standard	Happiness	0.88 (0.17)	0.67 (0.14)	0.9 (0.12)	0.77 (0.14)
	Sadness	0.44 (0.10)	0.52 (0.20)	0.49 (0.14)	0.57 (0.20)
	Surprise	0.44 (0.16)	0.46 (0.19)	0.41 (0.15)	0.44 (0.22)
	Fear	0.28 (0.18)	0.36 (0.21)	0.24 (0.16)	0.25 (0.15)
	Disgust	0.53 (0.21)	0.51 (0.18)	0.47 (0.14)	0.46 (0.14)
	Anger	0.57 (0.25)	0.51 (0.26)	0.31 (0.18)	0.34 (0.15)
Communicate	Happiness	0.82 (0.20)	0.66 (0.17)	0.84 (0.13)	0.73 (0.13)
	Sadness	0.46 (0.18)	0.48 (0.17)	0.54 (0.21)	0.47 (0.19)
	Surprise	0.52 (0.20)	0.53 (0.17)	0.54 (0.14)	0.57 (0.20)
	Fear	0.42 (0.25)	0.45 (0.23)	0.29 (0.13)	0.34 (0.18)
	Disgust	0.55 (0.15)	0.54 (0.18)	0.64 (0.14)	0.63 (0.16)
	Anger	0.61 (0.22)	0.63 (0.18)	0.38 (0.24)	0.42 (0.17)
Mirror	Happiness	0.85 (0.18)	0.73 (0.14)	0.93 (0.11)	0.74 (0.15)
	Sadness	0.53 (0.19)	0.63 (0.13)	0.53 (0.19)	0.56 (0.19)
	Surprise	0.55 (0.21)	0.50 (0.15)	0.62 (0.19)	0.51 (0.18)
	Fear	0.45 (0.26)	0.29 (0.14)	0.34 (0.21)	0.3 (0.17)
	Disgust	0.5 (0.24)	0.46 (0.14)	0.41 (0.11)	0.48 (0.13)
	Anger	0.64 (0.27)	0.52 (0.11)	0.34 (0.19)	0.4 (0.14)
*Confidence*					
Standard	Happiness	7.52 (1.28)	6.92 (1.00)	7.64 (1.47)	7.09 (1.34)
	Sadness	6.01 (1.62)	6.45 (1.15)	6.16 (1.26)	6.59 (1.37)
	Surprise	6.04 (1.40)	6.19 (1.52)	6.68 (1.30)	6.78 (1.29)
	Fear	5.9 (1.24)	6.64 (1.37)	6.3 (1.30)	6.46 (1.39)
	Disgust	6.7 (1.37)	6.56 (1.42)	7.01 (1.31)	7.16 (1.22)
	Anger	6.68 (1.46)	6.93 (1.32)	6.7 (1.34)	6.77 (1.47)
Communicate	Happiness	7.05 (1.35)	6.78 (1.38)	7.4 (1.11)	7.01 (1.33)
	Sadness	6.29 (1.45)	6.57 (1.34)	6.26 (1.53)	6.68 (1.32)
	Surprise	6.36 (1.38)	6.57 (1.54)	6.96 (1.30)	7.35 (1.18)
	Fear	6.45 (1.23)	6.51 (1.53)	6.46 (1.30)	6.77 (1.17)
	Disgust	6.85 (1.43)	6.65 (1.50)	7.3 (1.12)	7.15 (1.15)
	Anger	6.94 (1.49)	6.82 (1.61)	6.73 (1.43)	7.15 (1.23)
Mirror	Happiness	7.27 (1.16)	7.2 (1.07)	7.91 (1.17)	7.32 (1.20)
	Sadness	6.3 (1.24)	6.47 (1.29)	6.49 (1.39)	6.63 (1.26)
	Surprise	6.68 (1.26)	6.68 (1.23)	6.98 (1.32)	7.07 (1.26)
	Fear	6.77 (1.34)	6.31 (1.43)	6.81 (1.29)	6.55 (1.46)
	Disgust	6.53 (1.50)	6.7 (1.10)	6.77 (1.20)	7.01 (1.14)
	Anger	6.93 (1.30)	6.75 (1.44)	6.64 (1.46)	7.11 (1.16)

**Figure 2:**
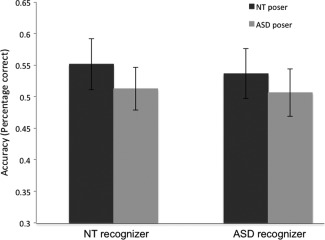
Expressions produced by NT posers were better recognized than those produced by ASD posers, regardless of ASD diagnosis of recognizer. Note that for illustration purposes raw data are plotted without the age covariate used in data analysis.

A main effect of Poser Group [*F*(1,24) = 5.53, *P* = 0.027, *ŋ* ^2^ = 0.187] indicated that NT posers (*M* = 0.54, SE = 0.02) were recognized better than ASD posers (*M* = 0.51, SE = 0.02; Fig. 2). A nonsignificant Poser Group × Emotion interaction suggested that this was the case across all emotional expressions, although the happiness condition was associated with a larger numerical difference between NT and ASD poser accuracy, as has been observed previously [Faso et al., 2014], although not consistently [Volker et al., 2009]. Emotion interacted significantly with Recognizer Group [*F*(5,120) = 3.01, *P* = 0.013, *ŋ* ^2^ = 0.112], suggesting that angry expressions were better recognized by NT individuals (*M* = 0.59, SE = 0.05) than by those with ASD (*M* = 0.36, SE = 0.05) [*t*(25) = 3.37, *P* = 0.002]. A nonsignificant main effect of Recognizer Group [*F*(1,24) = 1.344, *P* = 0.258, *ŋ* ^2^ = 0.053] indicated that this was not the case for other emotions.

**Figure 3:**
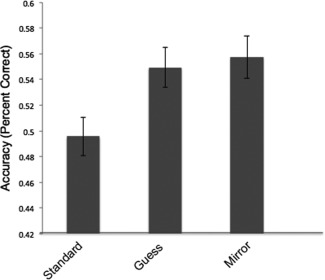
Expressions produced in the communicate condition and the mirror condition were recognized better than those produced in the standard posing condition, regardless of poser group (ASD or NT).

A significant main effect of Posing Condition [*F*(2,48) = 3.45, *P* = 0.040, *ŋ* ^2^ = 0.126] indicated that, across all participants and emotions, expressions produced in the standard posing condition (*M* = 0.49, SE = 0.01) were recognized less well than those produced in the communicate condition (*M* = 0.54, SD = 0.02) [*t*(26) = 5.2, *P* < 0.001] and the mirror condition (*M* = 0.53, SE = 0.02) [*t*(26) = 5.25, *P* < 0.001] (Fig. 3). A nonsignificant Poser Group × Posing Condition interaction indicated that the three posing conditions were equally effective for the ASD and NT posers.

A nonsignificant main effect of emotion indicated that expressions of different emotions were recognized equally well. Emotion interacted with posing condition, however, [*F*(10,240) = 2.62, *P* = 0.005, *ŋ* ^2^ = 0.098], suggesting that, while the posing conditions did not differentially affect recognizability of most expressions, fear expressions were better recognized when produced in the communicate (*M* = 0.38, SE = 0.03) [*t*(26) = 3.90, *P* = 0.001] and mirror (*M* = 0.34, SE = 0.03) [*t*(26) = 2.56, *P* = 0.018] than standard conditions (*M* = 0.28, SE = 0.03).

Due to the small number of female participants, gender effects could not be reliably investigated, but there were no significant effects of gender on ability to pose or recognize facial emotion. Further analysis of data without the inclusion of female stimuli or female recognizers produced the same pattern of results as reported with the full sample. It should be noted that the analysis without inclusion of female stimuli also led to the one ASD poser not meeting ADOS or AQ criteria for ASD being removed from analysis.

For confidence data, an identical Poser Group × Posing Condition × Emotion × Recognizer Group Analysis of Covariance, controlling for age, was conducted. This ANCOVA produced no significant results, suggesting confidence in recognition of emotional expressions was not influenced by ASD diagnosis of poser or recognizer, posing condition, or emotion being posed.

The recruitment of a representative sample of ASD and NT individuals meant that group analyses according to presence and absence of alexithymia was not possible. Similarly, while correlation analyses indicated that recognizer alexithymia was significantly associated with overall emotion recognition accuracy (*r* = −0.422, *P* = 0.028), alexithymia and autistic traits, measured by the AQ, were highly correlated (*r* = 0.667, *P* < 0.001). It is, therefore, impossible to determine whether the effects of ASD reported above are due to co‐occurring alexithymia or not. Given that alexithymia is prevalent in the ASD population, however, this does not detract from the main findings.

As a small number of individuals viewed expressions produced by themselves, it was possible to compare recognition of own and other expressions. Poser (self vs. other) × Emotion ANOVAs conducted separately in the ASD and control groups indicated that individuals with ASD recognized their own expressions (*M* = 0.63, SD = 0.15) better than the expressions of other individuals with ASD (*M* = 0.50, SD = 0.07) [*F*(1,5) = 7.79, *P* = 0.038]. There was also a main effect of emotion [*F*(5, 25) = 18.85, *P* < 0.001], indicating that happiness was recognized better than surprise (*P* = 0.021), fear (*P* = 0.019) and anger (*P* = 0.012), and fear was recognized less well than disgust (*P* = 0.010). Emotion interacted significantly with poser [*F*(5, 25) = 3.46, *P* = 0.016], indicating that individuals with ASD recognized their own expressions of happiness better than others’ (*P* < 0.001). In the typical group, despite the very small sample size, there was also a trend for individuals to recognize their own expressions (*M* = 0.81, SD = 0.11) more accurately than those of other individuals (*M* = 0.56, SD = 0.08) [*F*(1,3) = 8.80, *P* = 0.059], but poser did not interact with emotion [*F*(5,15) = 1.55, *P* = 0.234]. Again there was a main effect of emotion [*F*(5,15) = 5.85, *P* = 0.003], but no individual comparisons met the threshold for significance.

## Discussion

This study investigated the ability of individuals with and without ASD to recognize emotional facial expressions produced by NT and ASD posers. Results suggested that autistic expressions were more poorly recognized than NT expressions, regardless of recognizer group (ASD or NT), suggesting that representations of emotional expressions are atypical in ASD, and that these atypicalities are idiosyncratic, rather than systematic and shared, in the ASD population. Both groups of posers, however, produced more recognizable facial expressions when the communicative aspect of facial expression was emphasized, and when visual feedback was available.

Accuracy results indicated that, regardless of recognizer group, ASD expressions were recognized worse than NT expressions, in line with previous findings of “odd” and “unnatural” expressions in ASD [Faso et al., 2014; Volker et al., 2009]. While the effect size of the expression production impairment was relatively small, it should be noted that these expressions were produced while individuals were deliberately aiming to produce recognizable emotional expressions, and could sufficiently attend to and prepare for the task. Arguably impairment would be far greater in genuine interactions, in which individuals are required to process verbal information, attend to the interaction partner's expressions, and produce their own expressions without explicit instruction to do so. Interaction partners’ failure to correctly interpret ASD expressions may detrimentally affect the social interactions and relationships of individuals with ASD. Observers may disengage from individuals who do not express positive affect, or who express unexpected emotions in the context of the interaction, meaning atypical expression production is likely to have negative implications for ASD sociality.

Clearly, determining whether individuals with ASD also express emotion atypically in more ecologically valid situations, where expressions are spontaneous, remains a priority for future research. As real‐life interactions involve both spontaneous expressions (assumed to accurately indicate one's emotional state) and posed expressions (thought to be used by the poser to communicate attitudes and intentions to others), it is necessary to study both expression types, as each may be impaired in individuals with ASD. Further, once additional cognitive and emotional demands are involved in real‐life interactions, ASD expressions may differ even further from those of typical individuals.

Findings of poorly recognizable expressions in ASD are consistent with previous work [Macdonald et al., 1989; Volker et al., 2009]. The current findings extend existing results, however, by addressing the nature of the atypicalities. The fact that poser group and recognizer group did not interact suggests that both NT observers, and others with ASD, find it difficult to recognize autistic expressions. This suggests that individuals with ASD do not share common representations of emotional expressions. Rather, ASD expressions appear to be idiosyncratic, and therefore, even more problematic; individuals with ASD are likely to struggle to recognizably express emotion, regardless of whether they are interacting with ASD or NT individuals. As many individuals with ASD may prefer to interact with other members of the ASD population, this has particularly strong implications for the success of their social relationships. Notably, the possibility remains that subgroups with common representations of emotion exist within the ASD population (potentially characterized by varying levels of alexithymia, given its relationship with emotion processing in general). As the current sample is not large enough to determine whether this is the case, future research should prioritize investigation of this possibility.

When emotional expressions are produced atypically, this may be due to atypical internal representations of the physical features of emotional expressions, limited understanding of the communicative value of emotional expressions, or poor proprioceptive or motor feedback when producing emotional expressions. Here, expressions produced in a standard posing condition were recognized less well than those produced in either a communicative or visual feedback (mirror) condition, regardless of ASD diagnosis, suggesting both NT and ASD individuals benefit from the emphasis of the communicative function of emotional expressions, and from visual feedback, implicating imperfect representations of one's facial movements [Cook, Johnston, & Heyes, 2013]. Further, ASD expressions were recognized poorly across all posing conditions, suggesting that it is not the case that individuals with ASD simply do not understand that emotional expressions are informative to others (which would cause the ASD group to exhibit a greater improvement in the communicate condition than the NT group), nor that these individuals are less able than NT individuals to use proprioceptive feedback to produce emotional expressions (which would cause a greater improvement in the mirror condition). Instead, it seems that, despite visual feedback and explicit instruction to communicate emotion to others, individuals with ASD still produce emotional expressions that are difficult to interpret, implicating atypical internal representations of emotional expressions.

Individuals with ASD recognized expressions of anger less well than did NT recognizers, regardless of poser group (ASD vs. NT), but recognizer groups did not differ in their recognition of other emotions. Evidence regarding ability to recognize facial expressions has been mixed in existing ASD literature [Harms et al., 2010], but recent evidence suggests that co‐occurring alexithymia may explain deficits, where observed [Bird & Cook, 2013; Cook, Brewer, et al., 2013]. In this study, however, alexithymia and ASD symptomology were highly confounded, meaning it was impossible to determine whether alexithymia or ASD caused reduced ability to recognize facial anger. It is of note that alexithymia may also be responsible for the atypical expression production observed in the ASD group. Future work should, therefore, specifically match ASD and NT groups for alexithymia severity [Bird & Cook, 2013; Cook, Brewer, et al., 2013], in order to determine the independent effects of ASD symptomatology and alexithymia on expression production. Future work should also aim to determine whether ASD or alexithymia impairs ability to express emotion vocally or using with body posture [c.f. Heaton et al., 2012].

In conclusion, this study extends previous results concerning the ability of individuals with ASD to recognize and express emotion. Individuals with ASD produced atypical expressions, seemingly due to atypical representations of emotion, rather than simply having reduced comprehension of the use of emotional expressions, or awareness of their facial movements. These atypical representations also appear to be idiosyncratic, meaning members of the ASD populations may struggle to recognize emotional expressions produced by each other. These findings strongly suggest that increased attention should be paid to determining how representations of emotion in ASD may be trained, in order to improve social experiences for these individuals. Similarly, they emphasize the importance of those interacting with ASD individuals possessing awareness about the idiosyncratic nature of ASD emotional expression. Clearly, reduced ability to express one's emotion could be extremely detrimental to the quality of one's social interactions and relationships, emphasizing the need for further work into how expression production may be improved.

## References

[aur1508-bib-0001] American Psychiatric Association . (2013). Diagnostic and statistical manual of mental disorder (5th ed). Arlington, VA: American Psychiatric Publishing.

[aur1508-bib-0002] Ashwin, C. , Chapman, E. , Colle, L. , & Baron‐Cohen, S. (2006). Impaired recognition of negative basic emotions in autism: A test of the amygdala theory. Social Neuroscience, 1(3–4), 349–363. 18633799 10.1080/17470910601040772

[aur1508-bib-0003] Bagby, M. , Parker, J. D. A. , & Taylor, G. J. (1994). The twenty‐item Toronto alexithymia scale—I. Item selection and cross‐validation of the factor structure. Journal of Psychosomatic Research, 38(1), 23–32. 8126686 10.1016/0022-3999(94)90005-1

[aur1508-bib-0004] Baron‐Cohen, S. , Wheelwright, S. , Skinner, R. , Martin, J. , & Clubley, E. (2001). The autism‐spectrum quotient (AQ): Evidence from Asperger syndrome/high‐functioning autism, males and females, scientists and mathematicians. Journal of Autism and Developmental Disorders, 31, 5–17. 11439754 10.1023/a:1005653411471

[aur1508-bib-0005] Begeer, S. , Koot, H. M. , Rieffe, C. , Meerum Terwogt, M. , & Stegge, H. (2008). Emotional competence in children with autism: Diagnostic criteria and empirical evidence. Developmental Review, 28(3), 342–369.

[aur1508-bib-0006] Berthoz, S. , & Hill, E. L. (2005). The validity of using self‐reports to assess emotion regulation abilities in adults with autism spectrum disorder. European Psychiatry: The Journal of the Association of European Psychiatrists, 20(3), 291–288. 15935431 10.1016/j.eurpsy.2004.06.013

[aur1508-bib-0007] Bieberich, A. A. , & Morgan, S. B. (2004). Self‐regulation and affective expression during play in children with autism or Down Syndrome: A short‐term longitudinal study. Journal of Autism and Developmental Disorders, 34(4), 439–448. 15449519 10.1023/b:jadd.0000037420.16169.28

[aur1508-bib-0008] Bird, G. , & Cook, R. (2013). Mixed emotions: The contribution of alexithymia to the emotional symptoms of autism. Translational Psychiatry, 3(7), e285. 23880881 10.1038/tp.2013.61 PMC3731793

[aur1508-bib-0009] Brainard, D. H. (1997). The psychophysics toolbox. Spatial Vision, 10(4), 433–436. 9176952

[aur1508-bib-0010] Capps, L. , Kasari, C. , Yirmiya, N. , & Sigman, M. (1993). Parental perception of emotional expressiveness in children with autism. Journal of Consulting and Clinical Psychology, 61(3), 475–484. 8326050 10.1037//0022-006x.61.3.475

[aur1508-bib-0011] Cook, J. L. , Blakemore, S.‐J. , & Press, C. (2013). Atypical basic movement kinematics in autism spectrum conditions. Brain, 136(Pt 9), 2816–24. 23983031 10.1093/brain/awt208 PMC4017873

[aur1508-bib-0012] Cook, R. , Brewer, R. , Shah, P. , & Bird, G. (2013). Alexithymia, not autism, predicts poor recognition of emotional facial expressions. Psychological Science, 24(5), 723–732. 23528789 10.1177/0956797612463582

[aur1508-bib-0013] Cook, R. , Johnston, A. , & Heyes, C. (2013). Facial self‐imitation: Objective measurement reveals no improvement without visual feedback. Psychological Science, 24(1), 93–98. 23196637 10.1177/0956797612452568

[aur1508-bib-0014] Czapinski, P. , & Bryson, S. E. (2003). Reduced facial muscle movements in autism: Evidence for dysfunction in the neuromuscular pathway? Brain and Cognition, 51(2), 177–179.

[aur1508-bib-0015] Dawson, G. , Hill, D. , Spencer, A. , Galpert, L. , & Watson, L. (1990). Affective exchanges between young autistic children and their mothers. Journal of Abnormal Child Psychology, 18(3), 335–345. 2376657 10.1007/BF00916569

[aur1508-bib-0016] Dziobek, I. , Bahnemann, M. , Convit, A. , & Heekeren, H. R. (2010). The role of the fusiform‐amygdala system in the pathophysiology of autism. Archives of General Psychiatry, 67(4), 397–405. 20368515 10.1001/archgenpsychiatry.2010.31

[aur1508-bib-0017] Faso, D. J. , Sasson, N. J. , & Pinkham, A. E. (2015). Evaluating posed and evoked facial expressions of emotion from adults with autism spectrum disorder. Journal of Autism and Developmental Disorders, 1–15. 25037584 10.1007/s10803-014-2194-7

[aur1508-bib-0018] Greimel, E. , Schulte‐Rüther, M. , Kircher, T. , Kamp‐Becker, I. , Remschmidt, H. , Fink, G. R. , …, Konrad, K. (2010). Neural mechanisms of empathy in adolescents with autism spectrum disorder and their fathers. NeuroImage, 49(1), 1055–1065. 19647799 10.1016/j.neuroimage.2009.07.057

[aur1508-bib-0019] Grossman, R. B. , Edelson, L. R. , & Tager‐flusberg, H. (2013). Emotional facial and vocal expressions during story retelling by children and adolescents with high‐functioning autism. Journal of Speech, Language and Hearing Research, 56(3), 1035–1044. 10.1044/1092-4388(2012/12-0067) PMC3703874 23811475

[aur1508-bib-0020] Grynberg, D. , Chang, B. , Corneille, O. , Maurage, P. , Vermeulen, N. , Berthoz, S. , & Luminet, O. (2012). Alexithymia and the processing of emotional facial expressions (EFEs): Systematic review, unanswered questions and further perspectives. PLoS One, 7(8), e42429. 22927931 10.1371/journal.pone.0042429 PMC3426527

[aur1508-bib-0021] Halberstadt, A. G. , Denham, S. A. , & Dunsmore, J. C. (2001). Affective Social Competence. Social Development, 10(1), 79–119.

[aur1508-bib-0022] Harms, M. B. , Martin, A. , & Wallace, G. L. (2010). Facial emotion recognition in autism spectrum disorders: A review of behavioral and neuroimaging studies. Neuropsychology Review, 20(3), 290–322. 20809200 10.1007/s11065-010-9138-6

[aur1508-bib-0023] Heaton, P. , Reichenbacher, L. , Sauter, D. , Allen, R. , Scott, S. , & Hill, E. (2012). Measuring the effects of alexithymia on perception of emotional vocalizations in autistic spectrum disorder and typical development. Psychological Medicine, 42, 2453–2459. 22475181 10.1017/S0033291712000621

[aur1508-bib-0024] Hill, E. , Berthoz, S. , & Frith, U. (2004). Brief report: Cognitive processing of own emotions in individuals with autistic spectrum disorder and in their relatives. Journal of Autism and Developmental Disorders, 34(2), 229–235. 15162941 10.1023/b:jadd.0000022613.41399.14

[aur1508-bib-0025] Jessimer, M. , & Markham, R. (1997). Alexithymia: A right hemisphere dysfunction specific to recognition of certain facial expressions? Brain and Cognition, 34(2), 246–258. 9220088 10.1006/brcg.1997.0900

[aur1508-bib-0026] Kasari, C. , Sigman, M. , Mundy, P. , & Yirmiya, N. (1990). Affective sharing in the context of joint attention interactions of normal, autistic, and mentally retarded children. Journal of Autism and Developmental Disorders, 20(1), 87–100. 2139025 10.1007/BF02206859

[aur1508-bib-0027] Lane, R. D. , Sechrest, L. , Reidel, R. , Weldon, V. , Kaszniak, A. , & Schwartz, G. E. (1996). Impaired verbal and nonverbal emotion recognition in alexithymia. Psychosomatic Medicine, 58(3), 203–210. 8771618 10.1097/00006842-199605000-00002

[aur1508-bib-0028] Langdell, T. (1981). Face perception: An approach to the study of autism PhD Thesis, University of London.

[aur1508-bib-0029] Lindner, J. L. , & Rosén, L. A. (2006). Decoding of emotion through facial expression, prosody and verbal content in children and adolescents with Asperger's syndrome. Journal of Autism and Developmental Disorders, 36(6), 769–777. 16639533 10.1007/s10803-006-0105-2

[aur1508-bib-0030] Lord, C. , Risi, S. , Lambrecht, L. , Cook, E. H. , Leventhal, B. L. , DiLavore, P. C. , …, Rutter, M. (2000). The autism diagnostic observation schedule‐generic: A standard measure of social and communication deficits associated with the spectrum of autism. Journal of Autism and Developmental Disorders 30(3), 205–223. 11055457

[aur1508-bib-0031] Macdonald, H. , Rutter, M. , Howlin, P. , Rios, P. , Le Conteur, A. , Evered, C. , & Folstein, S. (1989). Recognition and expression of emotional cues by autistic and normal adults. Journal of Child Psychology and Psychiatry, and Allied Disciplines, 30(6), 865–877. 10.1111/j.1469-7610.1989.tb00288.x 2592470

[aur1508-bib-0032] McDonald, P. W. , & Prkachin, K. M. (1990). The expression and perception of facial emotion in alexithymia: A pilot study. Psychosomatic Medicine, 52(2), 199–210. 2330392 10.1097/00006842-199003000-00007

[aur1508-bib-0033] Pelli, D. G. (1997). The VideoToolbox software for visual psychophysics: Transforming numbers into movies. Spatial Vision, 10(4), 437–442. 9176953

[aur1508-bib-0034] Schilbach, L. , Timmermans, B. , Reddy, V. , Costall, A. , Bente, G. , Schlicht, T. , & Vogeley, K. (2013). Toward a second‐person neuroscience. The Behavioral and Brain Sciences, 36(4), 393–414. 23883742 10.1017/S0140525X12000660

[aur1508-bib-0035] Snow, M. E. , Hertzig, M. E. , & Shapiro, T. (1887). Expression of Emotion in Young Autistic Children. Journal of the American Academy of Child and Adolescent Psychiatry, 26(6), 836–838. 2448285 10.1097/00004583-198726060-00006

[aur1508-bib-0036] Stagg, S. D. , Slavny, R. , Hand, C. , Cardoso, A. , & Smith, P. (2014). Does facial expressivity count? How typically developing children respond initially to children with autism. Autism, 18(6), 704–711. 24121180 10.1177/1362361313492392

[aur1508-bib-0037] Swart, M. , Kortekaas, R. , & Aleman, A. (2009). Dealing with feelings: Characterization of trait alexithymia on emotion regulation strategies and cognitive‐emotional processing. PLoS One, 4(6), e5751. 19492045 10.1371/journal.pone.0005751 PMC2685011

[aur1508-bib-0038] Volker, M. A. , Lopata, C. , Smith, D. A. , & Thomeer, M. L. (2009). Facial encoding of children with high‐function autism spectrum disorders. Focus on Autism and Other Developmental Disabilities, 24(4), 195–204.

[aur1508-bib-0039] Wechsler, D. (1997). Wechsler Adult Intelligence Scale (3rd ed). San Antonio, TX: Psychological Corp.

[aur1508-bib-0040] Wechsler, D. (1999). Wechsler Abbreviated Scale of Intelligence. San Antonio, TX: Psychological Corp.

[aur1508-bib-0041] Weimer, A. K. , Schatz, A. M. , Lincoln, A. , Ballantyne, A. O. , & Trauner, D. A. (2001). “Motor” impairment in asperger syndrome: Evidence for a deficit in proprioception. Journal of Developmental & Behavioral Pediatrics, 22(2), 92–101. 11332785 10.1097/00004703-200104000-00002

